# Low Testosterone Among Males in Opioid Agonist Therapy and Its Association With Fatigue and Psychological Distress

**DOI:** 10.1210/jendso/bvaf114

**Published:** 2025-06-28

**Authors:** Jørn Henrik Vold, Elinor Chelsom Vogt, Anne Taraldsen Heldal, Fatemeh Chalabianloo, Karl Trygve Druckrey-Fiskaaen, Else-Marie Løberg, Paal Methlie, Kjell Arne Johansson, Lars Thore Fadnes

**Affiliations:** Department of Addiction Medicine, Haukeland University Hospital, Bergen N-5021, Norway; Department of Global Public Health and Primary Care, University of Bergen, Bergen N-5020, Norway; Division of Psychiatry, Haukeland University Hospital, Bergen N-5021, Norway; Department of Medicine, Haukeland University Hospital, Bergen N-5021, Norway; Department of Addiction Medicine, Haukeland University Hospital, Bergen N-5021, Norway; Department of Addiction Medicine, Haukeland University Hospital, Bergen N-5021, Norway; Department of Global Public Health and Primary Care, University of Bergen, Bergen N-5020, Norway; Norwegian Research Center for Agonist Treatment of Substance use Disorders, Department of Addiction Medicine, Haukeland University Hospital, Bergen N-5021, Norway; Department of Addiction Medicine, Haukeland University Hospital, Bergen N-5021, Norway; Department of Global Public Health and Primary Care, University of Bergen, Bergen N-5020, Norway; Division of Psychiatry, Haukeland University Hospital, Bergen N-5021, Norway; Department of Clinical Psychology, University of Bergen, Bergen N-5020, Norway; Department of Medicine, Haukeland University Hospital, Bergen N-5021, Norway; Department of Clinical Science, University of Bergen, Bergen N-5021, Norway; Department of Addiction Medicine, Haukeland University Hospital, Bergen N-5021, Norway; Department of Global Public Health and Primary Care, University of Bergen, Bergen N-5020, Norway; Department of Addiction Medicine, Haukeland University Hospital, Bergen N-5021, Norway; Department of Global Public Health and Primary Care, University of Bergen, Bergen N-5020, Norway

**Keywords:** opioid substitution treatment, opioid-induced hypogonadism, substance use disorder, testosterone, fatigue, psychological distress

## Abstract

**Context:**

Low testosterone levels are often considered an adverse effect of chronic opioid use. However, the prevalence of low testosterone levels and the association between testosterone levels and clinical symptoms among males receiving opioid agonist therapy (OAT) remain unclear.

**Objective:**

To biochemically investigate the prevalence of low testosterone levels (free testosterone ≤0.22 nmol/L) and the association between free testosterone levels and symptoms of fatigue, measured using the 3-item Fatigue Severity scale, and psychological distress, measured using the 10-item Hopkins Symptoms Checklist.

**Design and Setting:**

Prospective cohort study based on data collected between May 2017 and May 2024 from males receiving OAT recruited from OAT clinics at Haukeland University Hospital, Bergen, Norway.

**Participants:**

295 males aged 23 to 71 years, with a mean OAT duration of 8 years.

**Results:**

A total of 236 (80%) of participants had low testosterone levels at baseline, and 108/148 (73%) had low testosterone levels in 2 consecutive measurements. No association was found between free testosterone and fatigue (baseline: coefficient: −1.0, 95% confidence interval: −6.4; 4.5, Δscore/year: 0.8, −5.2; 6.8) or between free testosterone and psychological distress (baseline: 0.4, −0.3; 1.0, Δscore/year: −0.1, −0.3; 0.2).

**Conclusion:**

Eighty percent of males receiving OAT had low testosterone levels. However, testosterone levels were not associated with symptoms of fatigue or psychological distress. These findings should be interpreted with caution due to suboptimal power and potential influence of confounding. Further research is warranted to clarify the clinical significance of biochemical threshold values for low testosterone levels and their relationship to specific clinical symptoms in the OAT population.

Hypogonadism is often considered an adverse effect of long-term opioid use and has been reported in up to 63% of patients using opioids, with the highest prevalence among those with chronic opioid use [1-3]. The diagnosis of hypogonadism is generally assigned to males who exhibit consistent symptoms and signs of testosterone deficiency, along with unequivocally low serum testosterone levels [4]. Symptoms of testosterone deficiency include low libido; erectile dysfunction; and less specific symptoms such as fatigue, irritability, depressed mood, poor concentration, reduced physical performance, and sleep disturbance [4]. Among patients receiving long-acting opioids such as buprenorphine or methadone in opioid agonist therapy (OAT), these symptoms are common. Most of these patients report substantial levels of psychological distress and fatigue—higher levels than those detected in the general population [5-7]. Given the growing evidence of a globally increasing OAT population with more extensive physical and mental comorbidities [8, 9], it can be clinically challenging to differentiate symptoms of testosterone deficiency from those caused by other factors, such as substance withdrawals or intoxications, anabolic steroids, mental disorders, and infectious diseases. Therefore, research on how testosterone levels, as well as the types and doses of OAT medications, are associated with clinical symptoms is needed. This research could improve quality of life and retention in treatment, facilitate earlier and more appropriate treatment of testosterone deficiency, and protect against the adverse effects of long-term opioid use.

Opioids act on the hypothalamic-pituitary-gonadal axis and decrease the pituitary production of LH, primarily by reducing the pulsatility of GnRH secretion from the hypothalamus. This decreased LH secretion suppresses testosterone secretion from the testes [10]. A meta-analysis of 800 patients using opioids and 1969 controls found that patients using opioids had half the testosterone levels compared to the controls [11]. Long-acting opioids, such as methadone and buprenorphine, may suppress GnRH even more than shorter-acting opioids like heroin, likely due to cumulative suppression of the GnRH [12]. In 2 randomized controlled trials among patients receiving opioid analgesics for chronic noncancer pain, testosterone replacement therapy was effective in improving body composition, pain sensitivity, and sexual function in those with hypogonadism [13, 14]. However, among males receiving OAT, few studies—primarily cross-sectional studies with small samples—have investigated testosterone deficiency and its associations with clinical symptoms [15-19]. Although these studies have identified low testosterone levels, no large cohort studies have been conducted to examine the prevalence of testosterone deficiency using repeated measurements of testosterone levels in the OAT population. Additionally, there is a lack of research exploring the associations between testosterone deficiency, substance use, and clinical symptoms over time.

Testosterone deficiency is detected biochemically by measuring serum (s)-total testosterone levels [4]. However, these levels can vary based on laboratory methods, and there is currently no consistent threshold for defining testosterone deficiency [4]. Calculating free testosterone is recommended in cases of altered SHBG or borderline s-total testosterone levels [4] and can be estimated using different validated formulas. However, there is no defined reference interval for free testosterone, leading to variations in estimated values depending on the method used [4]. Among patients receiving OAT, where low testosterone levels are expected and concurrent clinical symptoms from other comorbid disorders may complicate the diagnostic assessment, knowledge of the thresholds for low testosterone levels using s-total testosterone and free testosterone according to guidelines [4] is essential. This knowledge can enhance diagnostic assessment and promote treatment for testosterone deficiency in this population.

The aim of this study was to investigate the prevalence of low testosterone levels using calculated free testosterone, assess the thresholds for low testosterone levels using calculated free testosterone and s-total testosterone, and identify the longitudinal association between free testosterone levels and symptoms of psychological distress and fatigue among males receiving OAT, adjusted for clinical and sociodemographic factors.

## Materials and Methods

### Data Sources

**Figure 1.**
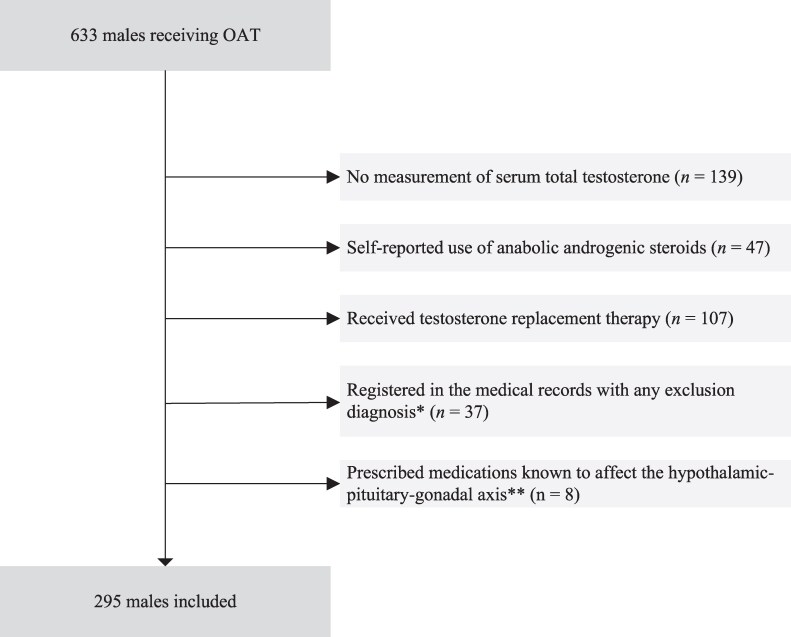
Flow chart of study sample. * Exclusion diagnoses (according to the International Classification of Diseases and Related Health Problems, 10th Revision codes); see Supplementary Material 1. ** Medications known to affect the hypothalamic-pituitary-gonadal axis: glucocorticosteroids, statins, anticorticosteroids (including ketoconazole), spironolactone, antiandrogens, aromatase inhibitors, and GnRH analogs. Abbreviations: OAT, opioid agonist therapy; SUD, substance use disorder.

We used data from nested cohorts from the INTRO-HCV and ATLAS4LAR studies in Bergen, Norway [20], approved by Helse Vest and the Regional Ethical Committee. The study was performed in accordance with the Declaration of Helsinki, Norwegian regulations, and relevant guidelines. It was reviewed and approved by the Regional Ethical Committee for Health Research (REK) West, Norway (reference number: 2017/51/REK West and REK South-East #155386, dated 29.03.2017/23.09.2020). Each patient provided written informed consent before enrolling in the study. Data were collected from May 2017 to May 2024. All participants were males over 18 years old, were recruited from OAT outpatient clinics, and were receiving OAT during the study period. All participants met the criteria for opioid dependence syndrome according to the International Classification of Diseases and Related Health Problems, 10th Revision. In the following chronological order, we excluded males who (1) did not have their s-total testosterone measured, (2) self-reported the use of anabolic androgenic steroids, (3) received testosterone replacement therapy, (4) were registered in the medical records with any of the defined exclusion diagnoses (see Supplementary Material 1 [21]), or (5) were prescribed medications known to affect the hypothalamic-pituitary-gonadal axis less than 1 year before recruitment to the study (Fig. 1).

### Data Collections

All consenting males were surveyed with at least 1 annual health assessment for sociodemographic factors, substance use, fatigue, and psychological distress. Blood samples were also collected annually. The data were collected by trained research nurses and stored in a health register using electronic data collection software, CheckWare (CheckWare AS, Trondheim, Norway). Clinical data on attainment, substance use, housing status, exclusion diagnoses, and prescribed medications were collected from the electronic medical records. Data on clinical symptoms and signs specific to hypogonadism (such as very small testes and loss of body hair) were not available [4]. A total of 295 participants were recruited for the study. Of those, 148 males completed 2 health assessments. The mean time from the first (baseline) to the second health assessment was 2.1 years, with a SD of 1.2 years.

### Biochemical Measurements of Hypothalamic-pituitary-gonadal Axis

Venous blood samples were collected during the health assessments and analyzed at the Department of Medical Biochemistry and Pharmacology at Haukeland University Hospital (accredited by ISO-standard 15189) for testosterone, albumin, SHBG, and LH in serum. Testosterone was measured using liquid chromatography with tandem mass spectrometry (analytic variation: 6%). LH (RRID: AB_2756388, coefficient of variation 7-8% at 10 IU/L; lower limit of quantitation 0.1 IU/L) and SHBG (RRID: AB_2819251, coefficient of variation 7% at 60 nmol/L; lower limit of quantitation 2 nmol/L) were measured using Siemens Immulite 2000 XPi, employing validated reagents approved. Albumin was measured using photometry (analytic variation: 3%). All blood samples were drawn before 12:00 Pm, although they should preferably be drawn before 10:00 Am, as testosterone levels can be 10% to 15% lower when drawn between 10:00 Am and 12:00 Pm [22]. However, most patients have delayed sleeping patterns, and they usually visit the OAT clinics shortly after waking up due to opioid withdrawals. Therefore, extending timing to 12:00 Pm was clinically assumed to be close to a reasonable adaption for blood samples for people with more regular sleeping patterns. Free testosterone was calculated using Vermeulen's formula, based on measured values of s-total testosterone, albumin, and SHBG (Supplementary Material 2 [21]). Vermeulen's formula is a simple and reliable index of bioavailable testosterone [23]. According to the Endocrine Society, we defined a low testosterone level as free testosterone ≤0.22 nmol/L [24]. We assessed the relationship between free testosterone and s-total testosterone using our laboratory's threshold for s-total testosterone, <6.7 nmol/L. Similarly, we defined the reference interval for LH from 0.8 to 7.6 IU/L and SHBG from 13 to 71 nmol/L according to our laboratory's thresholds.

### Measuring Fatigue and Psychological Distress

We measured fatigue using the 3-item Fatigue Severity Scale (FSS-3) and psychological distress using the 10-item Hopkins Symptoms Checklist (SCL-10). The FSS-3 questionnaire is validated for patients with substance use disorder, demonstrating excellent validity and reliability [25]. The SCL-10 questionnaire is validated in a Norwegian population and is widely used for clinical and epidemiological purposes [7, 26, 27]. The FSS-3 items were answered on a Likert scale ranging from 1 (no fatigue) to 7 (worst fatigue), indicating the fatigue level. We calculated the FSS-3 sum score by adding the points from the 3 items (range: 3-21 points). The SCL-10 questionnaire, which measures psychological distress, scored each item on 4 levels from “not bothered at all” (item score = 1) to “extremely bothered” (item score = 4). We generated the mean item score by summing the 10-item scores and dividing by the number of items completed. A total of 162 patients conducted 1 FSS-3 measurement, and 74 patients completed 2 FSS-3 measurements, resulting in a total of 310 FSS-3 measurements from 236 patients. Similarly, 143 patients conducted 1 SCL-10 measurement, and 141 completed 2 SCL-10 measurements amounting to 425 SCL-10 measurements from 284 patients.

### Definitions of Other Study Variables

“Frequent substance use” was defined as the use of any substance classes at least once a week during the past year preceding the annual assessment. The substance classes include alcohol, illicit stimulants (amphetamines and cocaine), benzodiazepines, cannabis, and opioids (excluding OAT medications). Patients who used substances less than weekly or did not use substances at all during the past year were categorized as having “no frequent use of substance.” The OAT dose ratio was calculated to compare the dose and type of OAT medication used. Following the World Health Organization's standard, we calculated the daily dose of received OAT opioids as the ratio between the received dose per day divided by the expected daily mean dose of OAT opioids (buprenorphine 18 mg, buprenorphine-naloxone 18/4.5 mg, methadone 93 mg, or morphine 350 mg) [25].

### Statistical Analyses

We used Stata/SE 17.0 (StataCorp, College Station, TX, USA) for descriptive and regression model analyses, and Sankeymatic (sankeymatic.com) to generate the Sankey diagram. IBM SPSS version 26.0 (IBM, Chicago, IL, USA) was utilized for expectation-maximization calculations. The threshold for statistical significance was set at *P* < .05 for all analyses.

We considered any missing values of the substances used as “missing at random” when performing the expectation-maximization algorithm. Missing values were identified in 10% of the substance variables (amphetamine, alcohol, benzodiazepines, cannabis, cocaine, and opioids), and all were replaced with estimated values. The expectation–maximization algorithm for computing data iteratively performed maximum likelihood estimation in the presence of latent variables [28], which is recommended for optimizing the mixed regression models.

We performed linear mixed model regression analyses to evaluate the association between free testosterone (exposure variable) and both the FSS-3 sum score and SCL-10 mean score (outcome variables). These analyses were adjusted for frequent substance use, age, having children (yes/no), OAT medication, and OAT dose ratio (handled as “silent” exposure variables) at baseline, as well as their association with changes in the scores reported at the second health assessment. Apart from the free testosterone variable, the other exposure variables were kept constant at the baseline level to predict the levels and changes in the outcome variables. We generated Δfree testosterone to investigate whether the changes in free testosterone between the health assessments were associated with the outcome variables. Both free testosterone and Δfree testosterone variables were treated as continuous exposure variables. To explore whether these exposure variables predicted changes in outcomes, interactions between these variables and time were added to the model. All available FSS-3 and SCL-10 measurements were included. A random intercept fixed slope model was used, with the estimator set to restricted maximum likelihood. Time was defined as years from baseline. Subgroup analyses (sensitivity analyses) were performed for FSS-3 and SCL-10 score outcomes by (1) stratifying the males into “low testosterone level” and “no low testosterone level” groups based on their baseline free testosterone levels and (2) including only those who had “low testosterone level” on both health assessments.

Post hoc power calculations for the longitudinal linear mixed model analyses were conducted based on previously reported mean sum score for the FSS-3 (14, SD: 5) and mean score for the SCL-10 (2.2, SD: 0.8) in patients with substance use disorders [29, 30]. Assuming lower testosterone levels among males receiving OAT, we estimated a 15% increase in the FSS-3 and SCL-10 scores for those with low testosterone levels and a 15% decrease for those with no low testosterone levels, compared to males with SUDs. This yielded anticipated mean scores of 16 and 12 for the FSS-3 and 2.5 and 1.9 for the SCL-10, respectively. One-sample power calculations with α = .05 and 1-β = .9 indicated a minimum sample size of 60 participants for the FSS-3 and 62 for the SCL-10. To account for potential confounding, these estimates were multiplied by a factor of 1.5, resulting in adjusted sample size requirements of 120 for the FSS-3 and 124 for the SCL-10.

## Results

### Characteristics of the Study Sample at Baseline

The mean age of the males was 44 years (SD: 10). Among them, 138 (47%) had children, and 147 (50%) had primary school listed as their highest level of education (Table 1). In the year leading up to the first health assessment, 131 (51%) had used cannabis, 91 (36%) had used benzodiazepines, 78 (30%) had used alcohol, 72 (28%) had used stimulants (amphetamines or cocaine), and 40 (16%) had used illicitly acquired opioids at least weekly. In the past 30 days, 76 (37%) participants had injected substances. The mean duration for which the males had received OAT was 8 years, with a SD of 5 years. The mean FSS-3 sum score was 14 (SD: 6), and the mean SCL-10 score was 2.1 (SD: 0.8). The mean values of SHBG and albumin were 60 nmol/L (SD: 29) and 45 g/L (SD: 3), respectively (Supplementary Material 3 [21]). No males had SHBG levels below the reference interval.

**Table 1.** Characteristics of patients at baseline (n = 295)

Age (years), n (%)	
18-25	5 (2)
25-40	106 (36)
40-60	161 (55)
≥ 60	23 (8)
Mean (SD)	44 (10)
Educational attainment, n (%) (n = 293)	
Not completed primary school	10 (3)
Primary school (9 years)	137 (47)
High school (12 years)	126 (43)
College or university	20 (7)
OAT medication, n (%) (n = 295)	
Methadone	103 (35)
Buprenorphine-based	186 (63)
Morphine or others	6 (2)
Children, n (%)	138 (47)
Number of years in opioid agonist therapy, mean (SD) (n = 216)	8 (5)
Injected substances past 30 days, n (%) (n = 203)	76 (37)
Unstable housing past 30 days*^a^*, n (%) (n = 294)	37 (13)
OAT dose ratio, mean (SD) (n = 294)	0.9 (0.5)
Frequent substance use during past year*^b^*, n (%) (n = 256)	
Alcohol	78 (30)
Cannabis	131 (51)
Benzodiazepines	91 (36)
Opioids (not OAT)	40 (16)
Stimulants (amphetamine or cocaine)	72 (28)
Mean age for the onset of substance use, mean (SD) (n = 238)	13 (3)
Body mass index, kg/m^2^, mean (SD) (n = 222)	26 (5)
Free testosterone*^c^* ≤ 0.22 nmol/L	236 (80)

Abbreviations: OAT, opioid agonist therapy.

^a^“Unstable housing status” was defined as living in a homeless shelter or with family or friends at any time during the 30 days leading up to the health assessment. “Stable housing status” was defined as having owned or rented housing or being incarcerated during the 30 days leading up to the health assessment.

^b^The number of patients who had used substances at least weekly during the past year leading up to baseline.

^c^Free testosterone calculated by Vermeulen's formula based on measured values of serum total testosterone, serum albumin, and serum SHBG.

### Testosterone Levels at Baseline and the Second Health Assessment

**Figure 2.**
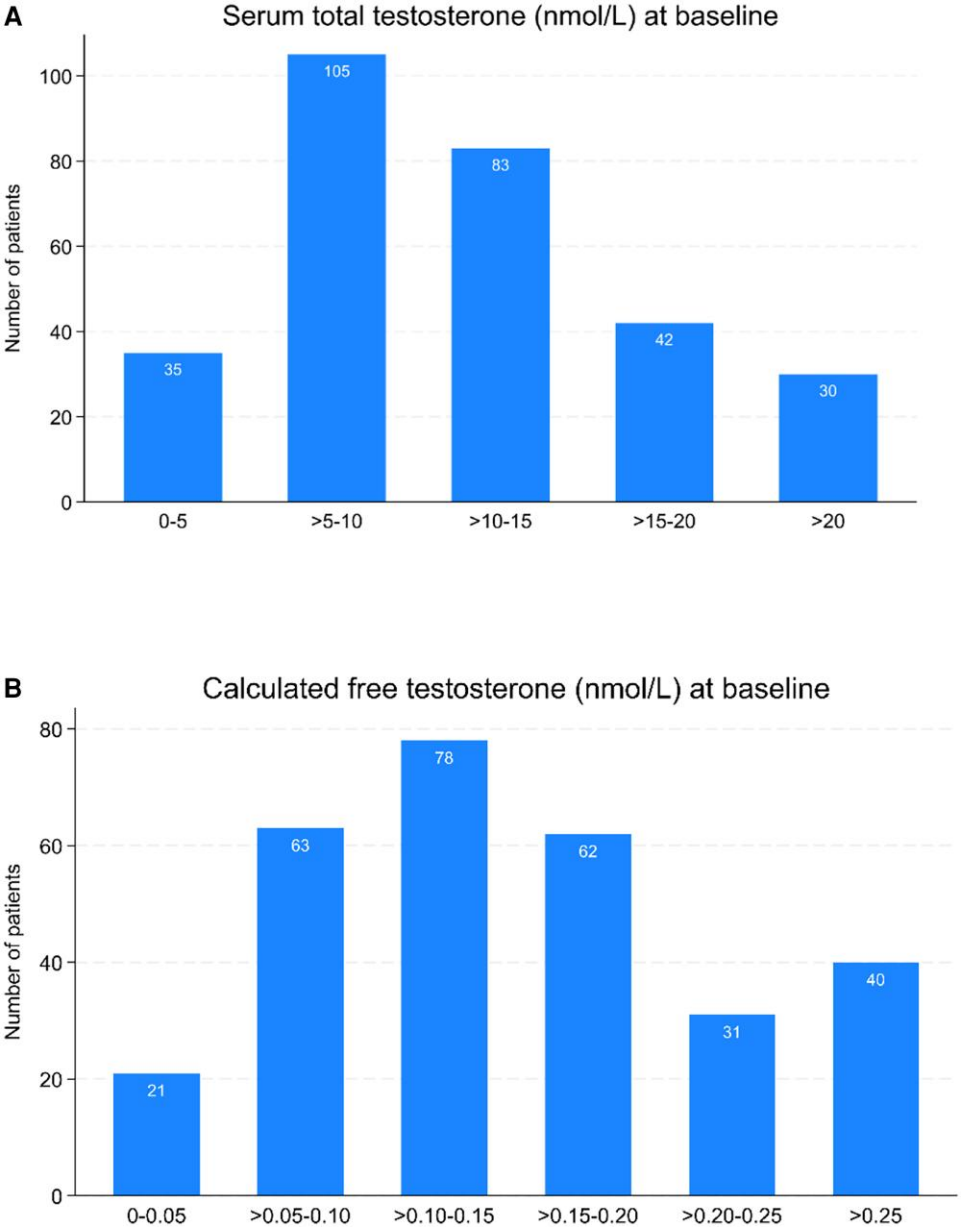
The proportion of males stratified by serum total testosterone (A) and calculated free testosterone (B) at baseline.

**Figure 3.**
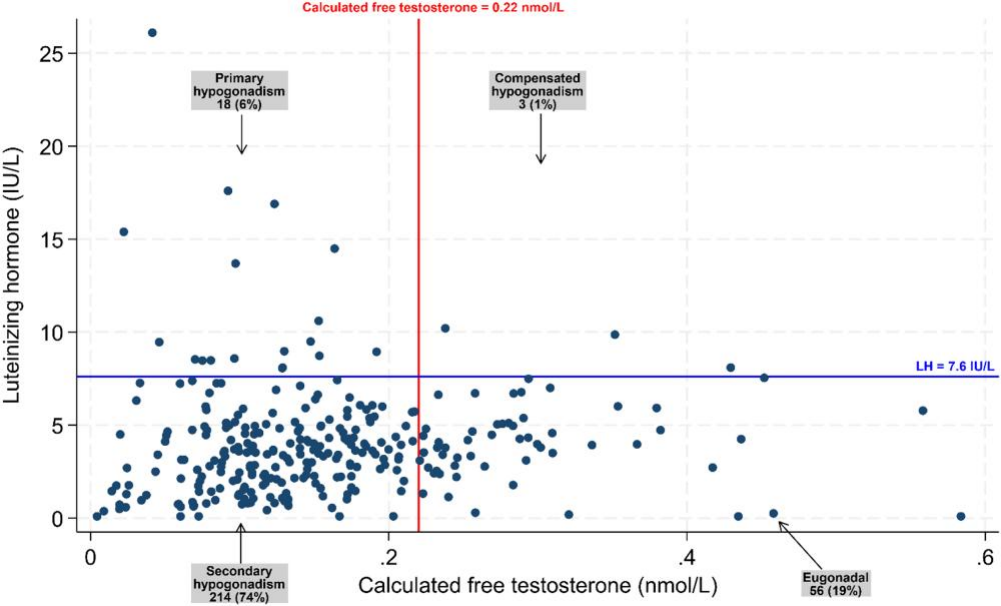
Box plot of the relationship between LH and calculated free testosterone at baseline. The box plot displays data from males who had both calculated free testosterone and LH values recorded at baseline (n = 291; 4 out of 295 males lacked LH measurements). The results are categorized into 4 classes based on calculated free testosterone and LH levels. A total of 214 males (74%) were likely to have secondary hypogonadism (calculated free testosterone ≤ 0.22 nmol/L and LH < 7.6 IU/L), 56 (19%) males were classified as eugonadal (calculated free testosterone > 0.22 nmol/L and LH < 7.6 IU/L), 3 (1%) males were likely to have compensated hypogonadism (calculated free testosterone > 0.22 nmol/L and LH ≥ 7.6 IU/L), and 18 (6%) males were likely to have primary hypogonadism (calculated free testosterone ≤ 0.22 nmol/L and LH ≥ 7.6 IU/L).

**Figure 4.**
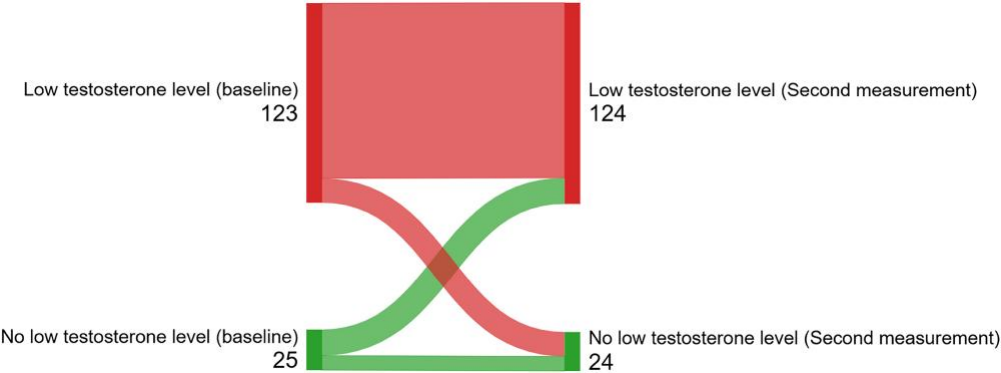
The number of males with “no low testosterone level” and “low testosterone level” at baseline and at the second measurement. The figure displays the number of males with 2 repeated testosterone measurements stratified by the results “low testosterone level” and “no low testosterone level” at baseline and second health assessment (n = 148). “Low testosterone level” was defined as calculated free testosterone ≤ 0.22 nmol/L and “no low testosterone level” was defined as calculated free testosterone > 0.22 nmol/L. Abbreviation: OAT, opioid agonist therapy.

At baseline, the mean total testosterone was 11.6 (SD: 7.4) nmol/L (Fig. 2A), while free testosterone was 0.16 (SD: 0.15) nmol/L (Fig. 2B). A total of 236 (80%) males had low testosterone levels based on calculated free testosterone at baseline, and of these, 214 (74%) had LH within or below reference interval (Fig. 3). Among the 73 (25%) males with s-total testosterone < 6.7 nmol/L, 65 (89%) had LH within or below the reference interval (Supplementary Material 4 [21]), and all had free testosterone ≤ 0.22 nmol/L at baseline. There were 148 (63%) males who completed 2 health assessments, and 108 (73%) of these males had free testosterone ≤ 0.22 nmol/L at both health assessments (Fig. 4).

### The Association Between Free Testosterone and Fatigue and Psychological Distress

No association was found between the FSS-3 sum score and free testosterone at baseline [coefficient: −1.0 (95% confidence interval: −6.4;4.5)] or from baseline to the second health assessment [Δsum score per year: 0.8 (−5.2;6.8)] (Table 2). Furthermore, free testosterone was not associated with the SCL-10 mean score either at baseline [0.4 (−0.3;1.0)] or between the health assessments (Δmean score per year: −0.1 [−0.3;0.2)]. “Low testosterone level” (free testosterone ≤ 0.22 nmol/L) vs “no low testosterone level” (free testosterone > 0.22 nmol/L) at baseline was not associated with symptoms of fatigue or psychological distress either at baseline or from baseline to the second health assessment (Supplementary Material 5 [21]). No association between symptoms of psychological distress or fatigue and free testosterone levels was found among those with low testosterone level in both health assessments (Supplementary Material 6 [21]).

**Table 2.** The association between free testosterone levels and the FSS-3 sum score and SCL-10 mean score

	FSS-3 coefficient (95% CI) (n = 236, number of observations: 310)				SCL-10 coefficient (95% CI) (n = 284, number of observations: 425)			
	Unadjusted		Adjusted		Unadjusted		Adjusted	
	Baseline	Δ sum score per year	Baseline	Δ sum score per year	Baseline	Δ mean score per year	Baseline	Δ mean score per year
Time (per year)	—	−0.6 (−1.1;−0.1)	—	−0.9 (−4.5;2.7)	—	−0.1 (−0.1;0.0)	—	0.1 (−0.3;0.5)
Free testosterone (nmol/L)	−0.7 (−5.8;4.5)	—	−1.0 (−6.4;4.5)	*—*	0.3 (−0.3;0.9)	—	0.4 (−0.3;1.0)	—
ΔFree testosterone (nmol/L)	—	−0.3 (−5.7;5.0)	—	0.8 (−5.2;6.8)	—	−0.1 (−0.3;0.2)	—	−0.1 (−0.3;0.2)

The table displays unadjusted and adjusted linear mixed model analyses of the association between free testosterone levels and the FSS-3 sum score and the SCL-10 mean score at baseline, as well as their change from baseline to the second health assessment (displayed as change in FSS-3 sum score and SCL-10 mean score per year, ie, time trend). For the FSS-3 sum score analyses, 236 patients who had conducted at least 1 health assessment, including FSS-3 and blood sample measurements, were included. Among these 236 patients, 74 patients had 2 FSS-3 and blood sample measurements, representing the time trend analysis from baseline to the second health assessment. For the SCL-10 mean score analyses, 284 patients who had conducted at least 1 health assessment, including SCL-10 and blood sample measurements, were included. Among these 284 patients, 141 patients had 2 SCL-10 and blood sample measurements, representing the time trend analysis from baseline to the second health assessment.

Abbreviations: CI, confidence interval; FSS-3, 3-item Fatigue Severity Scale; SCL-10, 10-item Hopkins Symptoms Checklist.

## Discussion

The present study found that 8 out of 10 males receiving OAT had low testosterone levels based on calculated free testosterone, with most of these having LH within or below the reference interval. The percentage of patients with low testosterone levels at baseline declined to 73% after confirmatory testing. More patients met the threshold for low testosterone levels when calculating free testosterone using Vermeulen's formula compared to using s-total testosterone alone. Despite the high prevalence of fatigue and psychological distress symptoms, no association was found between testosterone levels and these symptoms. The trend analysis of FSS-3 and subgroup analyses should be interpreted with caution due to suboptimal statistical power and the potential influence of confounding factors.

Although 80% of males receiving OAT had low testosterone levels based on calculated free testosterone at baseline, other studies have reported varying frequencies of hypogonadism, with prevalence rates ranging between 28% and 86% [10, 32-38]. A meta-analysis that included 3250 subjects using opioids for chronic pain or OAT (mainly methadone) reported a prevalence of hypogonadism of 63% [3]. The broad range of reported prevalence of hypogonadism among males using opioids can potentially be attributed to differing diagnostic thresholds for defining low testosterone levels and the use of unstandardized testing methods for analyzing and calculating testosterone levels. By applying a threshold of s-total testosterone <6.7 nmol/L [31], the prevalence of low testosterone levels was reduced by 69% in the present study. Free testosterone is considered a more accurate measure of circulating testosterone available for testosterone receptors and correlates better with clinical signs of hypogonadism among males than s-total testosterone [32]. In males with conditions that alter SHBG, including obesity, aging, HIV, use of anticonvulsants, and liver cirrhosis/hepatitis, which represented a third of our study population, calculating free testosterone concentrations is recommended [4]. However, few studies have evaluated testosterone levels by calculating free testosterone among patients receiving OAT [11], and no recognized threshold for low testosterone levels using free testosterone exists. Thus, further research is needed to determine the clinical significance of specific threshold values defining low testosterone levels in the OAT population.

Testosterone concentrations exhibit significant diurnal and day-to-day variations. Therefore, confirmatory morning measurements are important to increase their specificity and precision. In the present study, the prevalence of males with low testosterone levels declined from 80% at baseline to 73% in the second measurement. In total, 12% (15/123) of the males with baseline testosterone concentration in the biochemically low range had normal testosterone concentration on the repeated measurement. This proportion was substantially lower than the results in other studies evaluating repeated measurements of testosterone concentrations in randomly selected males aged 30 to 79 years [24, 33]. These results indicate that testosterone levels are persistently low among patients receiving OAT, and compared with the general population, the diurnal and day-to-day variation is less pronounced for most patients. This raises questions about whether repeated testosterone measurements are required to confirm the low testosterone levels in the OAT population [4].

Although psychological distress and fatigue levels were consistently high compared to the general population in the present study [7], free testosterone levels were not associated with levels of psychological distress or fatigue in the OAT population. However, studies on patients receiving OAT have found significant associations between frequent use of benzodiazepines and amphetamines or cocaine and symptoms of fatigue and psychological distress [29, 30]. Substance use may mask the symptoms of testosterone deficiency and significantly delay the diagnostic assessment of hypogonadism for most patients [4]. Over the past decade, the extent of polysubstance use has steadily increased, even among patients receiving OAT [34]. Polysubstance use is a crucial factor contributing to increased comorbidities of mental and physical disorders, such as drug-related psychosis, hepatitis C virus infection, and HIV infection [35]. It is also an independent factor for nutritional deficiencies associated with nonspecific symptoms and signs of testosterone deficiency [35]. This challenge emphasizes the need to consider clinical symptoms and signs more specific to low testosterone levels in this population. Indeed, a recent study indicates a correlation between sexual symptoms (poor morning erections, decreased libido, and erectile dysfunction) and both s-total testosterone and free testosterone [36]. Other specific signs, such as gynecomastia and loss of body (axillary and pubic) hair, should also be assessed [4]. Therefore, when diagnosing opioid-induced hypogonadism in males receiving OAT, the clinical presentation of specific symptoms of testosterone deficiency should be considered, followed by measurements of testosterone levels.

In some cases, males screened for testosterone deficiency in this study could be completely asymptomatic despite having detected testosterone deficiency. For these individuals, detected testosterone deficiency could cause unnecessary health concerns, and subsequent assessments are not recommended [4]. However, in the present study, only 3 males reported no symptoms of fatigue and psychological distress (lowest scores) at baseline. Recent studies in this population have also found significant fluctuations in symptoms of low testosterone levels over time [29, 30], posing a significant clinical challenge in distinguishing symptoms potentially related to testosterone deficiency from those caused by other factors over time, as well as asymptomatic periods. Thus, our results support measuring testosterone levels in symptomatic individuals, preferably those with persistent symptoms specific for testosterone deficiency.

Two randomized controlled trials [13, 14] and several retrospective and prospective studies have been conducted to assess the efficacy of testosterone replacement therapy in males using opiates (both patients with cancer pain and those with chronic noncancer pain). However, few studies include patients receiving OAT, where opioid doses are significantly higher and physical and mental comorbidities are usually more pronounced than in patients with chronic pain [4, 13, 14]. Transdermal gels, patches, and injections have all been utilized and evaluated using validated instruments measuring pain, sexual function, and psychological well-being [37]. Studies have demonstrated that testosterone replacement therapy in individuals with hypogonadism improves sexual function—including libido, erectile dysfunction, and sexual activity—compared to placebo [13, 14]. Additionally, testosterone replacement therapy has been associated with improvements in pain sensitivity, body composition, and quality of life [38-40]. However, the benefits and concerns of long-term use remain uncertain [4]. One meta-analysis did not find a significant difference in energy, mood, or cognitive improvement in hypogonadal males receiving testosterone replacement therapy compared with the general population [4]. Serious outcomes, such as infertility, androgen-sensitive cancers, heart failure, myocardial infarction, and stroke, have been reported for long-term exposure to testosterone therapy [4]. These risks should be particularly considered for patients receiving OAT, where opioid therapy is lifelong. Given the lack of evidence regarding the benefits and risks of long-term testosterone therapy in this population, caution is warranted when screening for low testosterone levels, comorbidities, risk of misuse of testosterone, and interactions with illegal substances and clinical and biochemical responses to treatment [41].

To our knowledge, this study is the largest one to investigate testosterone levels and related symptoms in a heterogeneous OAT population. A strength of this study was the inclusion of hard-to-reach participants receiving opioid doses higher than those assessed in previous high-quality studies [13, 14]. Another strength was the prospective cohort design, which, other than randomized controlled trials, is the best design to investigate repeated testosterone measurements and the associations between testosterone levels and psychological distress and fatigue. Additionally, performing longitudinal linear mixed model analyses, compared to other statistical methods, effectively prevented false-positive associations and increased statistical power by handling missing data from all patients [42, 43]. This approach decreased the risk of random associations and confounding factors, particularly when the prevalence of low testosterone levels was high. One important limitation is that a relatively high proportion of participants lacked repeated measurements of testosterone, SCL-10, and FSS-3. As a result, the trend analysis of FSS-3 scores and the subgroup analyses of both FSS-3 and SCL-10 scores were underpowered and based on observational data that may be subject to residual confounding beyond the variables adjusted for. Another limitation is that most males recruited were excluded due to self-reported use of anabolic androgenic steroids or prescribed testosterone agents, which could lead to an underestimation of testosterone levels. A third limitation of this study was that the FSS-3 and SCL-10 questionnaires were self-reported and did not identify specific symptoms of low testosterone levels. According to guidelines [4], using specific questionnaires, such as the Hypogonadism Impact of Symptoms Questionnaire [44], the Derogatis Interview for Sexual Functioning [45], and the International Index of Erectile Function [46], is recommended for diagnosing hypogonadism. Therefore, the prevalence of low testosterone levels in the present study is likely to be higher than in those with the formal diagnosis of hypogonadism. However, unlike the specific questionnaires for symptoms of hypogonadism, the SCL-10 and FSS-3 questionnaires are validated for substance use disorder populations and the general Norwegian population, demonstrating excellent validity and reliability [7, 25-27]. A fourth limitation is that blood samples were drawn during the annual health assessment of males receiving OAT, not specifically from those with symptoms and signs of testosterone deficiency [4]. This could lead to an overestimation of the prevalence of low testosterone levels. A fifth limitation is that the calculated free testosterone by Vermeulen's formula provided an estimate, not necessarily an accurate value of the free fraction. The precision of the free fraction is particularly low when SHBG is low [23]. However, of the 443 blood samples of testosterone, albumin and SHBG included, only 2 SHBG measurements were below the reference interval of 13 to 70 nmol/L, which are unlikely to affect the results. Additionally, total testosterone measurements were performed using the liquid chromatography with tandem mass spectrometry method, a robust and validated method for measuring the s-total testosterone, ensuring reliable measurements for calculating free testosterone adjusted for binding proteins SHBG and albumin.

In conclusion, the present study demonstrated that nearly 8 out of 10 males receiving OAT had low testosterone levels, as determined by calculated free testosterone. Among those with baseline free testosterone concentration in the hypogonadal range, only 12% had normalized levels upon repeated measurement. Despite consistently high levels of symptoms of fatigue and psychological distress, free testosterone levels were not associated with these symptoms. These findings should be interpreted with caution due to suboptimal statistical power and the potential influence of confounding factors. Further research is warranted to clarify the clinical significance of the biochemical threshold values for low testosterone and their relationship to specific clinical symptoms in the OAT population.

## Data Availability

The datasets analyzed during the current study are not publicly available due to data protection requirements. However, anonymous data files can be provided on reasonable request from the corresponding author.

## Abbreviations

FSS-3: 3-item Fatigue Severity Scale
OAT: opioid agonist therapy
REK: Regional Ethical Committee for Health Research
SCL-10: 10-item Hopkins Symptom Checklist
